# A meta-model of low back pain to examine collective expert knowledge of the effects of treatments and their mechanisms

**DOI:** 10.21203/rs.3.rs-7844247/v1

**Published:** 2025-10-30

**Authors:** Jacek Cholewicki, Paul W Hodges, John M Popovich, Payam Aminpour, Steven A Gray, Angela S Lee, Alan Breen, Simon Brumagne, Jaap H van Dieën, Linda R Van Dillen, Thomas E Dreisinger, Manuela L Ferreira, Steven Z George, Christine M Goertz, Jan Hartvigsen, Julie A Hides, Damian Hoy, Gregory N Kawchuk, Bart W Koes, Ralph Kothe, Helene M Langevin, Diane Lee, Jeffrey C Lotz, G. Lorimer Moseley, Heidi Prather, N. Peter Reeves, Shirley Sahrmann, Rob J Smeets, Laura S Stone, Johan W.S Vlaeyen, Jeffrey C Wang, Sherri Weiser

**Affiliations:** Michigan State University; University of Queensland; Michigan State University; Thermo Fisher Scientific (United States); Michigan State University; Michigan State University; Bournemouth University; KU Leuven; Vrije Universiteit Amsterdam; Washington University School of Medicine in St. Louis; Therapy Advisors; The University of Sydney; Duke University; Duke University; University of Southern Denmark; Griffith University; The University of Sydney; University of Alberta; Erasmus MC; Schön Klinik Hamburg Eilbek; National Institutes of Health; Diane Lee & Associates; University of California, San Francisco; University of South Australia; Hospital for Special Surgery; Sumaq Life LLC; Washington University School of Medicine in St. Louis; Maastricht University; University of Minnesota; KU Leuven; University of Southern California; New York University Langone Medical Center

## Abstract

**Purpose:**

Low back pain (LBP) is a complex, multifactorial condition with diverse contributors across biopsychosocial domains. Although personalized treatment is advocated, clear guidance on tailoring interventions is lacking. To help address this gap, we synthesized expert knowledge on treatment effectiveness and underlying mechanisms using a systems-based, collaborative modeling approach.

**Methods:**

Twenty-nine experts from diverse disciplines created individual fuzzy cognitive maps (FCMs) to represent their understanding of factors affecting pain, disability, and quality of life (QoL), along with treatment mechanisms. These maps were aggregated into a meta-model comprising 142 Components and 1,161 weighted Connections. Centrality was used to identify the most central domains of the meta-model. Simulations with the meta-model based on expert knowledge 1) estimated the relative effectiveness of treatments on pain, disability, and QoL and 2) identified key Mediators and mediating Domains based on their relative contribution to mediating treatment effects.

**Results:**

Psychological, biomechanical, and social/contextual Domains were central to expert conceptualizations of LBP. Simulation indicated cognitive behavioral therapy was considered the most effective among all interventions. Most interventions were mediated by Components across multiple Domains, with psychological factors frequently serving as mediators. The conceptual meta-model underscored the complexity of LBP, reflecting both its multifactorial nature and the diversity of expert perspectives on factors related to treatment effectiveness.

**Conclusion:**

The developed meta-model provides a novel, systems-based representation of expert knowledge about LBP, enabling quantitative exploration of treatment effects and underlying mechanisms. This conceptual framework also offers a foundation for advancing research on multi-modal, personalized care.

## Introduction

Multiple biological, psychological, and social factors contribute to low back pain (LBP) [1–4]. This complexity likely underlies the limited progress in reducing the high prevalence of LBP and its impact on disability and quality of life (QoL) [5, 6]. No single treatment or simple solution alleviates LBP across all individuals. Heterogeneity in patient responses results in small-to-moderate average treatment effects frequently reported in clinical trials [7–10].

It is reasonable to consider a multimodal approach addressing the biological, psychological, and social contributors to LBP [11, 12]. However, systematic reviews show only marginal benefits of multimodal over unimodal treatments [13, 14]. A key limitation is the lack of clear guidance on how to tailor multimodal treatment for individual patients - a core principle of “personalized” medicine. Diverse opinions on how to implement personalization have further hindered progress [15–18].

Efforts to address LBP complexity and heterogeneity have included the use of machine learning and “big data” to identify patient phenotypes and match them with multimodal interventions specific to each phenotype [19, 20]. These efforts have yielded mixed results, highlighting the challenges of translating phenotypic classifications into effective, personalized care [21]. A critical step toward improving treatment personalization is the development of a thorough understanding of the factors driving LBP, how these factors interact, and the mechanisms of interventions for LBP. There have been calls for the development of comprehensive, state-of-the-art models of LBP to identify knowledge gaps, guide research, and better understand LBP dynamics [4, 22]. Chau et al. [4] describe such models as “conceptual representations, mental models, or patterns of knowledge” about LBP.

Systems science provides tools for addressing complex, multifactorial problems. One approach, “collaborative modeling”, integrates diverse interest holder perspectives and has been validated in environmental management to support decision-making [23, 24]. We applied this approach to examine individual expert opinions on LBP, treatment effectiveness and mechanisms [2]. Clinical and research experts identified key contributing factors and modeled their interactions using fuzzy cognitive maps (FCMs) [25]. These FCMs provided a quantitative description of how experts conceptualize LBP, i.e., mental models [2]. Here we aimed to: (i) aggregate individual FCMs into a single meta-model representing collective expert knowledge, and (ii) use this meta-model to simulate and compare treatment effectiveness and underlying mechanistic pathways, with the goal of informing future research on personalized LBP management.

## Methods

### Part 1: Building the meta-model

The meta-model was constructed by aggregating individual FCMs previously collected from experts in LBP. The detailed methodology for obtaining these FCMs is described elsewhere [2]. Briefly, 29 clinicians and researchers with expertise in LBP (e.g., publications, contributions to professional societies [2]) participated. They represented diverse disciplines (basic science (n = 7), chiropractic (n = 4), spine surgery (n = 2), physical medicine & rehabilitation (n = 2), physical/exercise therapy (n = 12), and psychology (n = 2)), including those active in research and clinical practice. Each participant completed a structured interview to construct an FCM using the Mental Modeler platform (www.mentalmodeler.org) [26]. These FCMs captured the participants’ conceptual understanding (mental model) of factors involved in LBP, their interactions, and the effects of various interventions on patient-reported primary Outcomes (Pain, Disability, QoL). Participants listed all relevant factors (Components), identified relationships (Connections) between them, and assigned Weights (from − 1 to 1) to indicate the direction and strength of each Connection. Participants also listed all treatments (Treatments/Interventions) they believed could affect Outcomes and mapped out the experts’ knowledge of pathways of their effects (Connections and Weights).

After refining and consolidating similar terms, a total of 147 unique Components from the 29 FCMs were categorized into ten Domains based on the International Classification of Diseases (ICD) framework [27]: (1) Outcomes, (2) Behavioral/Lifestyle, (3) Biomechanical, (4) Individual, (5) Comorbidities, (6) Tissue injury or pathology, (7) Psychological, (8) Nociceptive detection and processing, (9) Social/Work/Contextual factors, and (10) Treatment/Intervention. Further refinement of terms in the present study reduced the total to 142 Components (Online Resource 1 outlines minor changes from our previous study).

For this study, each participant’s FCM was transformed into a 142×142 adjacency matrix to standardize dimensions and to include the weighting of the connections between the 142 unique Components identified across all models. Instead of leaving missing values, Connections not specified by a participant were assigned a weight of zero, indicating the participant did not consider the connection meaningful. In the absence of data to weigh the credibility of individual FCMs, a simple averaging method, including zeros, was used to aggregate the matrices [28, 29]. The aggregated adjacency matrix was imported into Gephi 0.10.1 (https://gephi.org) [30] for visualization. Table 1 shows metrics computed to describe the meta-model’s structure.

### Part 2: Evaluating the relative effects of treatments

To evaluate the relative effects of treatments on pain, disability, and QoL, we conducted simulations using the meta-model and a custom Python-based software employing a sigmoid transfer function (PyFCM [31], Python Software Foundation, www.python.org). Each Treatment/Intervention was independently initialized to a state value of 1. The state of each Component was then iteratively updated by propagating the initial input through the meta-model network, based on Connection Weights and the transfer function, until all values converged to a steady state [32]. Final state values of the outcome Components (Pain, Disability, QoL) were then recorded. These simulation results should be interpreted in relative terms (i.e., ordinal ranking), as the chosen transfer function was selected to ensure model convergence rather than to represent exact relationships between Components [33].

### Part 3: Examining mechanisms mediating treatment effects

To explore the mechanisms underlying each treatment, we identified the Components and Domains that served as primary Mediators between treatments and outcomes. Although Treatments/Interventions may affect outcomes through complex pathways involving multiple intermediary Components, our analysis focused on first-level (direct) Connections from Treatments/Interventions to other Components to identify primary Mediators of treatment effects. For each Treatment/Intervention, the Component (excluding Outcomes) with the highest absolute Connection weight was the primary Mediator. To determine the most influential Domain mediating each treatment, we summed the absolute Weights of all direct Connections from Treatments/Interventions to Mediators within Domains. These sums were used to rank the Domains by their relative contribution to mediating treatment effects.

## Results

### Part 1: Building the meta-model

**Figure 1:**
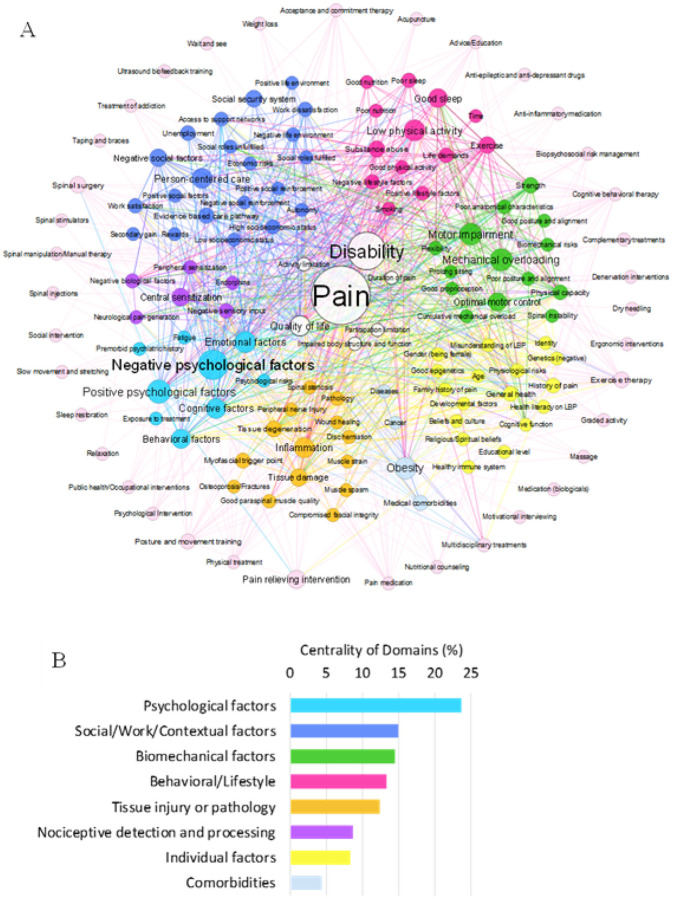
(A) Meta-model representing expert knowledge of LBP. Outcomes (Pain, Disability and Quality of Life) are shown in the center, while Treatments/Interventions are displayed around the periphery. Circle size is proportional to Component Centrality, and Colors indicate the ten Domains based on the ICD framework. (B) Relative Centrality of each Domain (excluding Outcomes and Treatments/Interventions), calculated as the sum of Centrality of all Components within the Domain, and expressed as a percentage of total model Centrality.

The meta-model had 142 Components including 3 representing the primary Outcomes (Pain, Disability, QoL) and 37 representing Treatments/Interventions (Table 2). There were 1,161 Connections (interactions) between Components, underscoring the complexity of LBP and the breadth of expert perspectives. The meta-model’s structure reflects the expert group’s collective understanding of LBP (Fig. 1A, also available in high resolution and as an adjacency matrix in Online Resources 2 and 3). For clarity, Outcomes are at the meta-model’s center with Treatments/Interventions at the periphery. Component and Connection colors represent the Domains. The size of each circle reflects the Component’s Centrality. The three Domains with the highest Centrality were Psychological, Social/Work/Contextual, and Biomechanical (Fig. 1B), indicating that Components and Connections within these Domains were most strongly emphasized by participants.

### Part 2: Evaluating the relative effects of treatments on outcomes

Simulation of treatment scenarios identified the most effective interventions based on collective expert opinion. Although treatment rankings varied slightly across Outcomes, treatments combined under the heading of cognitive behavioral therapy (CBT) were consistently ranked as the most effective for improving pain, disability, and QoL (Table 3). Pain medication was second for reducing pain, whereas exercise therapy ranked second for reducing disability and improving QoL. Other top-ranked treatments included physical treatment, posture and movement training, advice/education, and acceptance and commitment therapy, though their relative effectiveness varied by Outcomes. The least effective treatments were either proposed by few participants, perceived as having limited effectiveness or viewed as beneficial only for specific patient subgroups.

### Part 3: Examining the mechanisms mediating effects of treatments

There were 317 Connections between Treatments/Interventions and their Mediators (Online Resource 4). Table 4 lists the highest-ranked Mediator and the highest-ranked mediating Domain for each Treatment/Intervention. Generally, the highest-ranked Mediator belonged to the highest-ranked mediating Domain. However, there were exceptions. For example, the primary Mediator for spinal manipulation was reduced unhealthy expectations, beliefs, and perceptions concerning pain – Psychological Component – despite the Biomechanical Domain being highest ranked for this treatment.

**Figure 2:**
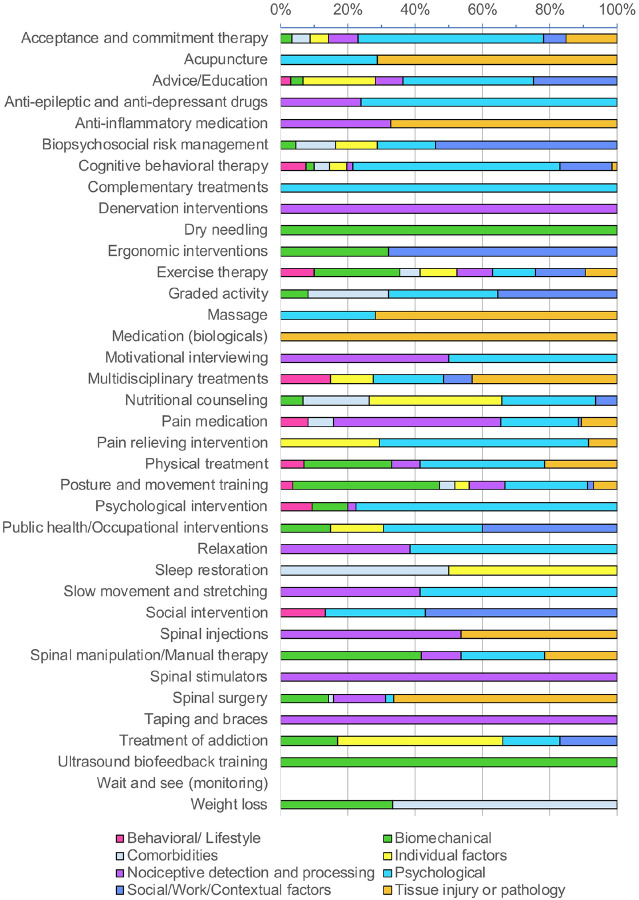
Relative contribution of each Domain to the mediation of treatment effects. For each Treatment/Intervention, the absolute Weights of Connections to Mediators were summed within each Domain and expressed as a proportion of the total Weight of all mediating Connections across Domains (excluding direct Treatment/Intervention-Outcome Connections).

Figure 2 illustrates the relative contribution of each Domain to mediation of treatment effects. For most treatments, Mediators spanned multiple Domains. For example, exercise therapy involved Mediators from all domains with a slight emphasis on Biomechanical Components. In contrast, interventions such as complementary treatments and denervation procedures had more focused pathways, with Mediators arising exclusively from a single Domain: Psychological and Nociceptive detection and processing, respectively.

## Discussion

This study developed a meta-model that synthesizes the diverse perspectives of a multidisciplinary group of LBP experts. The model reflects a shared view of LBP as a complex condition involving numerous contributing factors across eight Domains, a broad range of treatments, and many potential mechanisms by which treatments influence clinical outcomes. This meta-model offers a tool to inform future research to advance the development of personalized LBP care.

### Complexity of LBP

Constructed using a collaborative modeling approach, the meta-model reinforces the well-established understanding that LBP is highly complex [1, 3, 34] and that expert opinions differ, likely reflecting disciplinary backgrounds, research and clinical experiences [4]. A key advantage of our approach is that it synthesizes this diversity into a coherent framework. Unlike narrative or systematic reviews, which describe complexity qualitatively and propose strategies to address it [1, 3, 34], our meta-model enables simulations of hypothetical scenarios, offering a novel means to inform research on personalized LBP treatment.

Related efforts include a meta-model derived from perspectives of people with lived experience of LBP [35] and another focused on sacroiliac joint (SIJ) pain [36]. The patient-derived model was substantially simpler and emphasized biomechanical factors, contrasting expert meta-model’s emphasis on psychological factors. Patients favored non-surgical, non-pharmacological, physical treatments (e.g., exercise therapy, and slow movement and stretching), whereas the expert model identified CBT as the most effective treatment. The SIJ model was more biomechanically oriented and emphasized injections or surgery, although exercise was also recognized as beneficial [36].

### Relative effects of treatments

Simulations produced treatment rankings that broadly align with published clinical data [37, 38], supporting the effectiveness of interventions such as CBT [39], exercise therapy [40], acceptance and commitment therapy [41], counseling and education [42, 43], and physical therapy [44] for managing LBP. General agreement between the expert-derived model and evidence-based recommendations is reassuring. It also underscores the value of incorporating expert opinion into evidence-based practice, particularly in areas where high-quality empirical data are limited [45]. By synthesizing perspectives across multiple disciplines, this collaborative meta-model facilitates decision-making processes and offers a more balanced and comprehensive foundation for clinical guideline development than reliance on individual expert views alone [46–48].

Treatments combined under the heading of CBT were ranked the most effective treatments for pain, disability, and QoL. CBT’s effects in the meta-model were mediated primarily through psychological factors. Key mediators of CBT included emotional factors such as distress, anxiety and depression, which many consider to be main contributors to pain behavior [49–51]. CBT’s effectiveness across pain, disability and QoL is reasonable given they are interrelated to some extent [52].

### Mediators of treatment effects

The meta-model enabled investigation into how treatment effects are mediated to influence outcomes. Most treatments operated through Mediators spanning multiple Domains, suggesting the involvement of multiple mechanisms of action. Psychological Mediators appeared in the pathways of nearly all interventions, which is unsurprising given that the Psychological Domain exhibited the highest Centrality in our meta-model. These findings underscore the overlap in mechanistic pathways across treatments, which may help explain why combining interventions often yields limited additional benefit for LBP [13, 14]. Future research could use this model to examine specific mediating pathways and identify unique features to guide personalized treatment strategies.

### Limitations

Several limitations should be acknowledged. First, we treated missing Connections as zeroes. This assumes participants omitted them because they believed the connections had no effect. Although this approach reduces the influence of uncommon or idiosyncratic opinions, it may attenuate some plausible Connections unintentionally omitted. Second, this meta-model does not account for different presentations of LBP (e.g., different time courses and different diagnoses) and cannot reflect differences in treatment effectiveness based on patient phenotypes. Third, the participant group included a high proportion of physical therapists, potentially biasing the model toward that discipline’s perspectives. Fourth, although this study focused on expert input, incorporating perspectives from individuals with lived experience is increasingly recognized as essential in collaborative research. We have separately collected mental models from these individuals [35], which can be compared with the current expert-driven meta-model. Fifth, the Connection Weights are based entirely on expert opinion rather than empirical data. One application of this meta-model is to identify knowledge gaps and generate hypotheses to test these opinions. Sixth, despite extensive consultation and careful model aggregation [2], some terms or Domain assignments may not perfectly reflect participants’ original intentions. Seventh, simulation results are affected by the number of Connections between a treatment and its outcome. Each intervening Connection decreases the state of the subsequent Component based on the assigned weight - treatments with longer or more complex pathways might appear less effective than those with direct pathways to outcomes. Eighth, it is possible that knowledge of experts has increased or changed since the FCMs were collected.

### Future directions

A general objective of this work was to support the development of personalized LBP care. Progress towards this goal must overcome two barriers: (i) precise determination of an individual’s phenotype could be difficult because of the unique interplay among many contributing factors in each case, making phenotyping infeasible, and (ii) the possibility of tailoring intervention to match an individual phenotype could be limited, because current treatments for LBP are mediated through multiple overlapping pathways without the necessary precision. Nevertheless, this meta-model can help prioritize research efforts by: (i) identifying high-impact mediators and pathways to optimize treatment combinations for further evaluation, and (ii) highlighting key relationships (Connections) that require empirical data for their precise weighting.

Future model enhancements could include the following. First, additional FCMs could be included. Although it might be assumed that additional participants would strengthen the meta-model, it is not clear that > 30 provides additional value [53]. One exception would be the inclusion of under-represented disciplines that might provide additional treatments and alternative understanding of mechanisms. Second, “big data” could be sought to provide objective Weights for the Connections, as outlined in the theoretical framework by Huie et al. [54] and attempted by Zhu et al. [55]. Third, future work could convert this meta-model into a dynamic one [56, 57], capturing the time-dependency of treatment effects (e.g., acute vs. chronic LBP) and accounting for changes in certain factors during over the course of this condition. This approach was applied to investigate dynamics between opioid use and chiropractic care for chronic pain [58]. Fourth, repeating this modeling approach may reveal evolving views among experts and patients [59].

## Conclusion

This study presents a systems-based meta-model that synthesizes expert knowledge of LBP, offering a novel framework for exploring the relative effectiveness of treatments and their mechanisms. The model highlights the complexity of LBP, underscores the role of psychological factors, and identifies CBT as a broadly effective intervention across primary outcomes. Although the current model is not designed to establish causal relationships, it provides a valuable foundation for hypothesis generation, empirical testing, and the development of personalized LBP treatments. This model could be used for education and training, communicating with patients, and facilitating interdisciplinary discussion and collaboration.

## Supplementary Material

This is a list of supplementary files associated with this preprint. Click to download.

- ESM1MetamodelChanges.docx
- ESM2MetamodelHDFigure.svg
- ESM3MetamodelMatrix.xls
- ESM4TreatmentMediators.xlsx

## Figures and Tables

**Table 1:** Metrics describing structure of the meta-model.

Metric	Definition
Total Components (N)	Number of Components included the meta-model
Total Connections (C)	Total number of Connections in either direction included in the meta-model
Density (D)	Number of Connections as a proportion of the number of all possible Connections in both directions
Connections per Component	Average number of Connections in either direction per Component
Number of Driver Components	Total number of Components that only have outputs
Number of Receiver Components	Total number of Components that only have inputs
Number of Ordinary Components	Number of Components with both inputs and outputs
Complexity Score	Calculated as the ratio of Receiver/Driver Components and provides a measure of the degree to which effects of Drivers are considered
Centrality of Domains	Calculated as a sum of the absolute values of all Connections in and out of all Components classified into a given Domain

**Table 2:** Summary of meta-model parameters describing its structure.

Parameter	Meta-model value
Total Components (N)	142
Total Connections (C)	1161
Density (D)	0.058
Connections per Component	8.176
Number of Driver Components	50
Number of Receiver Components	0
Number of Ordinary Components	92
Complexity Score	0

**Table 3:** Simulation results, using the meta-model based on the experts’ knowledge of LBP, showing the relative effectiveness of Treatments/Interventions for each Outcome: Pain, Disability, and Quality of Life (QoL). Treatments/Interventions are ranked from most to least effective. Results are presented on an ordinal scale, as the numerical values reflect rankings only and should not be interpreted as interval magnitudes of effects.

Rank	Pain	Disability	Quality of Life
1	Cognitive behavioral therapy	Cognitive behavioral therapy	Cognitive behavioral therapy
2	Pain medication	Exercise therapy	Exercise therapy
3	Physical treatment	Advice/Education	Acceptance and commitment therapy
4	Exercise therapy	Physical treatment	Posture and movement training
5	Posture and movement training	Posture and movement training	Advice/Education
6	Acceptance and commitment therapy	Spinal manipulation/Manual therapy	Physical treatment
7	Advice/Education	Acceptance and commitment therapy	Spinal manipulation/Manual therapy
8	Spinal manipulation/Manual therapy	Spinal surgery	Nutritional counseling
9	Spinal surgery	Multidisciplinary treatments (biopsychosocial treatments)	Biopsychosocial risk management
10	Pain relieving intervention	Pain relieving intervention	Spinal surgery
11	Acupuncture	Biopsychosocial risk management	Pain relieving intervention
12	Massage	Treatment of addiction	Treatment of addiction
13	Multidisciplinary treatments (biopsychosocial treatments)	Graded activity	Sleep restoration
14	Anti-inflammatory medication	Psychological intervention	Acupuncture
15	Biopsychosocial risk management	Pain medication	Graded activity
16	Nutritional counseling	Nutritional counseling	Massage
17	Sleep restoration	Sleep restoration	Multidisciplinary treatments (biopsychosocial treatments)
18	Graded activity	Weight loss	Pain medication
19	Treatment of addiction	Wait and see (monitoring)	Weight loss
20	Psychological intervention	Acupuncture	Psychological intervention
21	Spinal injections	Massage	Public health/ Occupational interventions
22	Anti-epileptic and anti-depressant drugs	Spinal stimulators	Anti-epileptic and anti-depressant drugs
23	Weight loss	Complementary treatments	Anti-inflammatory medication
24	Spinal stimulators	Anti-inflammatory medication	Spinal injections
25	Slow movement and stretching (e.g., yoga)	Anti-epileptic and anti-depressant drugs	Slow movement and stretching (e.g., yoga)
26	Complementary treatments	Public health/ Occupational interventions	Wait and see (monitoring)
27	Wait and see (monitoring)	Spinal injections	Spinal stimulators
28	Denervation interventions	Slow movement and stretching (e.g., yoga)	Complementary treatments
29	Public health/ Occupational interventions	Social intervention	Social intervention
30	Medication (biologicals)	Ergonomic interventions	Relaxation
31	Social intervention	Relaxation	Motivational interviewing
32	Taping and braces	Denervation interventions	Ergonomic interventions
33	Relaxation	Motivational interviewing	Denervation interventions
34	Ergonomic interventions	Ultrasound biofeedback training	Ultrasound biofeedback training
35	Motivational interviewing	Medication (biologicals)	Medication (biologicals)
36	Ultrasound biofeedback training	Taping and braces	Taping and braces
37	Dry needling	Dry needling	Dry needling

**Table 4:** Top-ranked Mediator Components and top-ranked mediating Domains for each Treatment/Intervention, listed alphabetically. In the event of ties, multiple Mediators or Domains are listed. The results are derived from the meta-model based on collective expert knowledge.

Treatment/ Intervention	Mediator	Direction of Effect	Mediating Domain
Acceptance and commitment therapy	Emotional (e.g., distress, anxiety, depression)	Decrease	Psychological
Acupuncture	Tissue damage	Decrease	Tissue injury or pathology
Advice/Education	Cognitive (e.g., expectations, beliefs, perceptions concerning pain)	Decrease	Psychological
Anti-epileptic and anti-depressant drugs	Emotional (e.g., distress, anxiety, depression)	Decrease	Psychological
Anti-inflammatory medication	Inflammation	Decrease	Tissue injury or pathology
Biopsychosocial risk management	Evidence based care pathway	Decrease	Social/Work/Contextual factors
Cognitive behavioral therapy	Emotional (e.g., distress, anxiety, depression)	Decrease	Psychological
Complementary treatments	Positive psychological factors	Increase	Psychological
Denervation interventions	Neurological pain generation	Decrease	Nociceptive detection and processing
Dry needling	Optimal motor control	Increase	Biomechanical
Ergonomic interventions	Negative life social factors	Decrease	Social/Work/Contextual factors
Exercise therapy	Negative biological factors (e.g., postural control, genetics, tissue damage, central sensitization) AND Good paraspinal muscle quality	Decrease Increase	Biomechanical
Graded activity	Autonomy	Increase	Social/Work/Contextual factors
Massage	Tissue damage	Decrease	Tissue injury or pathology
Medication (biologicals)	Inflammation	Decrease	Tissue injury or pathology
Motivational interviewing	Negative psychological factors AND Negative biological factors (e.g., postural control, genetics, tissue damage, central sensitization)	Decrease Decrease	Psychological AND Nociceptive detection and processing
Multidisciplinary treatments (biopsychosocial treatments)	Tissue damage	Decrease	Tissue injury or pathology
Nutritional counseling	Positive psychological factors	Increase	Individual factors
Pain medication	Negative sensory input	Decrease	Nociceptive detection and processing
Pain relieving intervention	Physiological risks	Decrease	Psychological
Physical treatment	Motor impairment	Decrease	Psychological
Posture and movement training	Cognitive (e.g., expectations, beliefs, perceptions concerning pain)	Decrease	Biomechanical
Psychological intervention	Emotional (e.g., distress, anxiety, depression)	Decrease	Psychological
Public health/ occupational interventions	General health	Increase	Social/Work/Contextual factors
Relaxation	Negative psychological factors	Decrease	Psychological
Sleep restoration	Overweight (obesity/BMI) AND Physiological risks	Decrease Decrease	Comorbidities AND Individual factors
Slow movement and stretching (e.g., yoga)	Emotional (e.g., distress, anxiety, depression)	Decrease	Psychological
Social intervention	Negative life social factors	Decrease	Social/Work/Contextual factors
Spinal injections	Neurological pain generation	Decrease	Nociceptive detection and processing
Spinal manipulation/Manual therapy	Cognitive (e.g., expectations, beliefs, perceptions concerning pain)	Decrease	Biomechanical
Spinal stimulators	Neurological pain generation	Decrease	Nociceptive detection and processing
Spinal surgery	Disc herniation	Decrease	Tissue injury or pathology
Taping and braces	Negative sensory input	Decrease	Nociceptive detection and processing
Treatment of addiction	Physiological risks	Decrease	Individual factors
Ultrasound biofeedback training	Optimal motor control	Increase	Biomechanical
Wait and see (monitoring)	N/A	N/A	N/A
Weight loss	Overweight (obesity/BMI)	Decrease	Comorbidities

## Data Availability

The data are provided in the Online Resource files.
